# Efficacy of Cellular Therapy for Diabetic Foot Ulcer: A Meta-Analysis of Randomized Controlled Clinical Trials

**DOI:** 10.1177/0963689717738013

**Published:** 2018-02-02

**Authors:** Ye Zhang, Hong Deng, Zhouping Tang

**Affiliations:** 1 Department of Neurology, Tongji Hospital, Tongji Medical College, Huazhong University of Science and Technology, Wuhan, Hubei, China

**Keywords:** diabetic foot, cell transplantation, meta-analysis, diabetes mellitus

## Abstract

Diabetes mellitus is a widely spread chronic disease with growing incidence worldwide, and diabetic foot ulcer is one of the most serious complications of diabetes. Cellular therapy has shown promise in the management of diabetic foot ulcer in many preclinical experiments and clinical researches. Here, we performed a meta-analysis to evaluate the efficacy and safety of cellular therapy in the management of diabetic foot ulcer. We systematically searched PubMed, MEDLINE, EMBASE, and Cochrane Library databases from inception to May 2017 for randomized controlled trials assessing the efficacy of cellular therapy in diabetic foot ulcer, and a meta-analysis was conducted. A total of 6 randomized controlled clinical trials involving 241 individuals were included in this meta-analysis. The results suggested that cellular therapy could help accelerating the healing of diabetic foot ulcer, presented as higher ankle-brachial index (mean difference = 0.17, 95% confidence interval [CI] = 0.11 to 0.23), higher transcutaneous oxygen pressure (standardized mean difference [SMD] = 1.43; 95% CI, 1.09– to 1.78), higher ulcer healing rate (relative risk [RR] = 1.78; 95% CI, 1.41 to 2.25), higher amputation-free survival (RR = 1.25; 95% CI, 1.11 to 1.40), and lower scale of pain (SMD = −1.69; 95% CI, −2.05 to −1.33). Furthermore, cellular therapy seemed to be safe, with no serious complications and low risk of short-term slight complications. Cellular therapy could accelerate the rate of diabetic foot ulcer healing and may be more efficient than standard therapy for diabetic foot treatment.

## Introduction

The prevalence of diabetes mellitus (DM) worldwide is gradually increasing these years and this figure is predicted to continue growing, resulting in more than 360 million patients with DM in 2030.^1^ Although it is manageable, diabetic foot ulcer is one of the most common and serious complications secondary to DM. Statistical data have indicated that 2% to 3% of patients with DM were suffering from active foot ulcer and a quarter of DM patients would develop foot ulcer throughout their lifetime.^2,3^ Diabetic foot ulcer not only affects the physical health of patients, preceding 85% of major lower limb amputations in patients with DM,^4^ but also has a significant effect on their social function and mental health.^5^ Furthermore, the patients and society have to bear the substantial financial burden from treatment and care of diabetic foot ulcer.^6^ The diabetic foot ulcer, in which neuropathy and peripheral vascular disease act as major pathogenic factors, is featured by the typical dysfunctions of wound healing, including coagulation, hemostasis, inflammation, proliferation, and remodeling.^7^ Nowadays, the conventional standard therapy for diabetic foot ulcer consists of glucose-level control, infection management, high pressure remission, and dressings. However, the effectiveness of conventional standard therapy is not satisfying enough. Even with comprehensive treatment programs, the cure rate of diabetic foot ulcer in 12 to 20 wk was only as low as 24% to 30%. More seriously for patients, they are at high risk of serious complications, such as cellulitis, osteomyelitis, amputation, and others.^8–10^

Cellular therapy, characterized by using cells from diverse sources, with self-renewing potential and multidifferentiation ability, has shown promise in the management of diabetic foot ulcer. Accumulating evidences from basic science studies and clinical trials have pointed out that cellular therapy could focus on multiple facets during diabetic foot ulcer healing through cell proliferation, vascularization, neurorestoration, inflammation regulation, exosomes synthesis, and others.^11,12^ Some clinical studies have demonstrated that cellular therapy represents an effective treatment for diabetic foot ulcer. However, reliable evidence on the clinical efficacy remains to be addressed. Therefore, the present study evaluates and synthesizes clinical evidence and aims to critically estimate the therapeutic efficacy of cellular therapy for diabetic foot ulcer compared to standard therapy.

## Research Design and Methods

### Search Strategy

An extensive literature search restricted to the English language was carried out up to May 2017 using the PubMed, MEDLINE, EMBASE, and Cochrane Library databases. The search terms we used were (stem cells, mononuclear cells [MNCs], and progenitor cells) and (diabetic foot, diabetic ulcer, and diabetic wound). In addition, we examined the reference list of all relevant articles.

### Selection Criteria

Publications were screened independently by 2 authors. Studies meeting the following criteria were included: (1) randomized controlled trials comparing cellular therapy with standard therapy conducted in humans, (2) patients with diabetic foot ulcer, (3) full articles reporting the clinical efficacy. Studies that carried out in animals, lacking standard therapy as controls or lacking sufficient data of interest, were excluded. Referring to duplicate publications, the latest or larger one was included in the analysis.

### Data Extraction and Quality Assessment

Effectiveness outcomes including ankle-brachial index (ABI), amputation-free survival (AFS), transcutaneous oxygen tension pressure (TcPO_2_), ulcer healing rate at 12 to 24 wk posttransplantation, and pain scales and the adverse events representing safety profile occurring during each trial were extracted from all the included studies by 2 authors independently.

The risk of bias of the included clinical trials was assessed in accordance with the modified Jadad rating scales.^13^ Randomization, blinding, and follow-up were rated as yes, no, and not reported.^13^

Discrepancies about literature search, study selection, data extraction, and quality assessment between the 2 authors were settled by discussion and consensus or determined by a senior author.

### Statistical Analysis

Extracted data were entered and processed by Stata statistical software, version 12.0 (StataCorp, College Station, TX, USA). In order to estimate the clinical efficacy, mean difference and 95% confidence intervals (CIs) were calculated for quantitative variables and relative risk, and 95% CIs were calculated for dichotomous variables. Two-sided tests and a significant heterogeneity level of *P* < 0.05 were used in all analyses. Heterogeneity was estimated by the *I* ^2^ statistics, with values of 25%, 50%, and 75% being considered low, moderate, and high heterogeneity, respectively. When high heterogeneity was present, weighted mean difference and random effects model were applied to minimize heterogeneity; otherwise, the fixed effects model was used. We also performed Egger’s intercept test and Begg’s rank correlation analysis to estimate publication bias and conducted sensitivity analysis to examine the reliability of outcomes.

## Results

### Literature Search

**Fig. 1.**
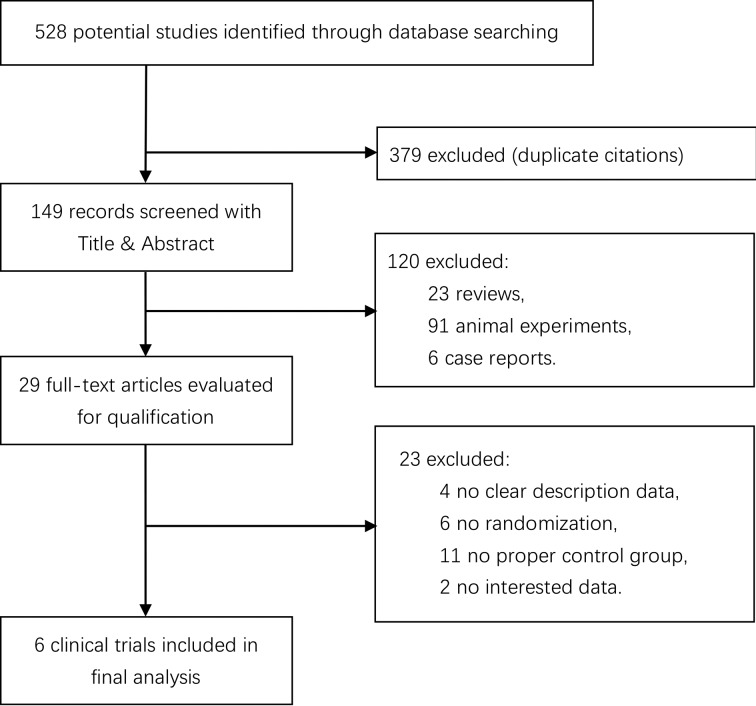
Flow chart of study selection.

The initial literature search yielded a total of 528 articles. After deletion of 379 duplicates, the remaining abstracts were carefully screened. One hundred twenty studies were excluded for various reasons such as reviews, animal experiments, and case reports. Of all the 29 remaining studies, 6 randomized controlled clinical trials meeting all criteria and providing a clear description data were selected.^14–19^ The screening process of the trials is shown in Fig. 1.

### Study Characteristics

After selection, 6 randomized controlled clinical trials involving 241 patients were included. Considering the cell type used in each study, 2 studies^14,19^ used mesenchymal stem cells from bone marrow or umbilical cord, 3 studies^15,17,18^ used MNCs from bone marrow or peripheral blood, and 1 study^16^ used bone marrow-derived mesenchymal stem cells (BMMSCs) and bone marrow-derived MNCs (BMMNCs). Details of the study characteristics are listed in Table 1.

**Table 1.** Study Characteristics of Included Studies.

Cellular Therapy/Standard Therapy								
Reference	Study Design	No. of Patients	Age^a^, Years	Diabetes Duration^a^, Years	Cell Type	Cell Dose	Transplantation Route	Follow-up
Dash et al. ^14^	RCT	3/3	40 ± 10	/	BMMSC	>10^6^/cm^2^	Muscle injection	12 wk
Huang et al. ^15^	RCT	23/24	71.1 ± 5.9/70.9 ± 6.0	12.9 ± 8.9/11.6 ± 8.0	PBMNC	3 × 10^9^	Muscle injection	12 wk
Lu et al. ^16^	RCT	37/37	63 ± 8/65± 10	10.3 ± 5.6/9.8 ± 5.0	BMMSC/BMMNC	9.6 ± 1.1× 10^8^	Muscle injection	24 wk
Mohammad-zadh et al. ^17^	RCT	7/14	63.5 ± 7.8/64.2 ± 7.8	14.2 ± 8.5/16.5 ± 8.7	PBMNC	1.5–2.0 × 10^7^/ site	Muscle injection	12 wk
Ozturk et al. ^18^	RCT	20/20	71.9 ± 9.2/70.8 ± 8.8	13.5 ± 8.5/12.8 ± 9.8	PBMNC	2.48 × 10^7^	Muscle injection	12 wk
Qin et al. ^19^	RCT	28/25	75 ± 3/73 ± 5	12.8 ± 7.2/13.1 ± 4.6	UCMSC	4.8–8.6 × 10^7^	Muscle injection	12 wk

Abbreviations: BMMSCs, bone marrow-derived mesenchymal stem cells; BMMNCs, bone marrow-derived mononuclear cells; PBMNCs, peripheral blood mononuclear cells; RCT, randomized controlled trials; SD, standard difference; UCMSCs, umbilical cord-derived mesenchymal stem cells.

^a^Values are presented in mean ± standard difference.

### Quality Assessment

As evaluated by modified Jadad rating scales,^13^ 3 included studies were high-quality randomized clinical trials.^14,16,18^ Quality assessment outcomes of all the included studies are presented in Table 2. All the included studies were described as randomized clinical trials, and 3 studies reported adequate sequence generation such as using random number table,^14^ randomization table,^16^ or internet-based system.^18^ Allocation concealment was described only in 1 study.^14^ One study claimed to be double-blinded, but details of blinding were not reported in this study.^16^ All studies described the details of loss to follow-up.

**Table 2.** Quality Assessment of the Included Studies.

Reference	Adequate Sequence Generation	Allocation Concealment	Blinding	Adequate Report on Loss to Follow-up	Total Score
Dash et al.^14^	Y	Y	N	Y	5
Huang et al.^15^	NR	NR	N	Y	3
Lu et al.^16^	Y	NR	NR	Y	5
Mohammad-zadh et al.^17^	NR	NR	N	Y	3
Ozturk et al.^18^	Y	NR	N	Y	4
Qin et al.^19^	NR	NR	N	Y	3

Abbreviations: N, No; NR, not reported; Y, Yes.

### ABI

**Fig. 2.**
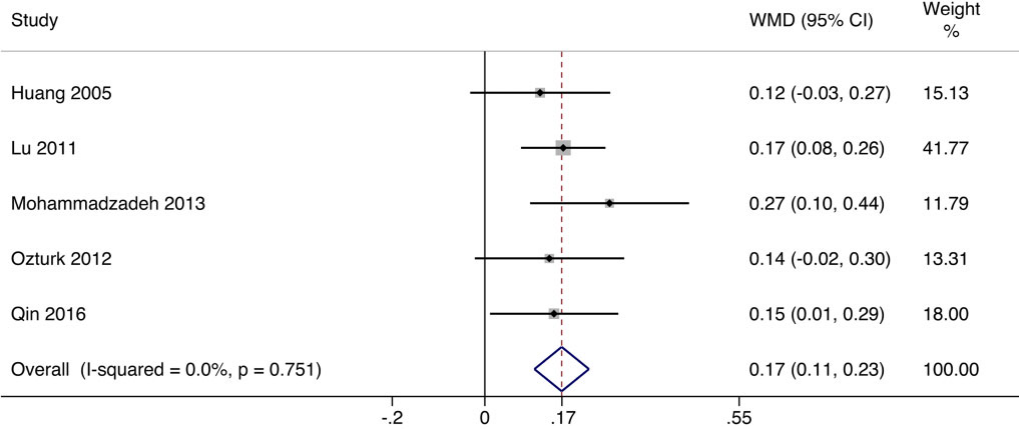
Forest plots for meta-analysis of ankle-brachial index (ABI) comparing cellular therapy with standard therapy. Area of the symbols for each study (square) is proportional to study weight. The pooled mean difference (fixed effects) and 95% confidence intervals are represented by the rhombus. Heterogeneity test across studies was significant (*I* ^2^ = 0.0%, *P* = 0.751). CI, confidence interval; WMD, weighted mean difference.

The ABI is defined as the ratio of the highest pressure detected by Doppler at the dorsalis pedis and posterior tibial arteries and the highest pressure at the brachial artery. The ABI provides such a great deal of information that it has become a routine measurement in the patients with diabetic foot ulcer. Five of the included studies^15–19^ involving 235 patients reported the ABI. Owing to no heterogeneity between studies (*I* ^2^ = 0.0%, *P* = 0.751), fixed effects model was applied in meta-analysis. The outcomes revealed that ABI was significantly raised by cellular therapy (mean difference = 0.17, 95% CI, 0.11 to 0.23; Fig. 2).

### Transcutaneous Oxygen Pressure (TcPO_2_)

**Fig. 3.**
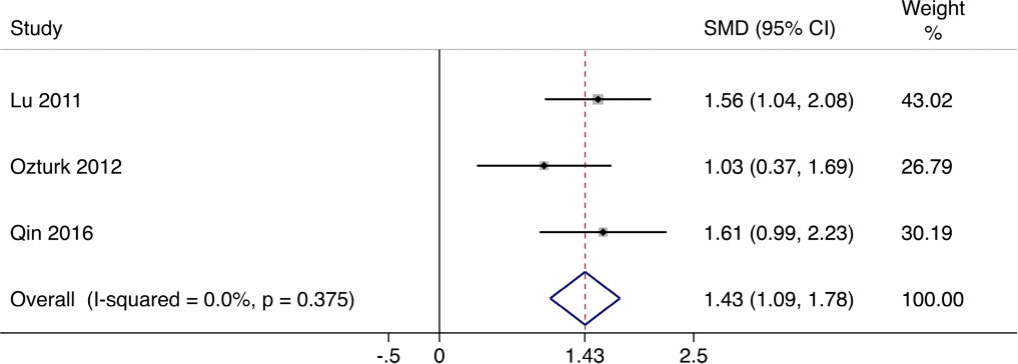
Forest plots for meta-analysis of TcPO_2_ comparing cellular therapy with standard therapy. Area of the symbols for each study (square) is proportional to study weight. The pooled standardized mean difference (fixed effects) and 95% confidence intervals are represented by the rhombus. Heterogeneity test across studies was significant (*I* ^2^ = 0.0%, *P* = 0.375). CI, confidence interval; SMD, standardized mean difference.

TcPO_2_ is known to be an indicator of the local microcirculation and the degree of ischemia and is valuable to predict healing at various levels of foot ulcer.^20,21^ In the patients treated with cellular therapy, the TcPO_2_ values significantly increased (standardized mean difference [SMD] = 1.43; 95% CI, 1.09 to 1.78) in the analysis of 3 studies^16,18,19^ (Fig. 3).

### Pain Scale

**Fig. 4.**
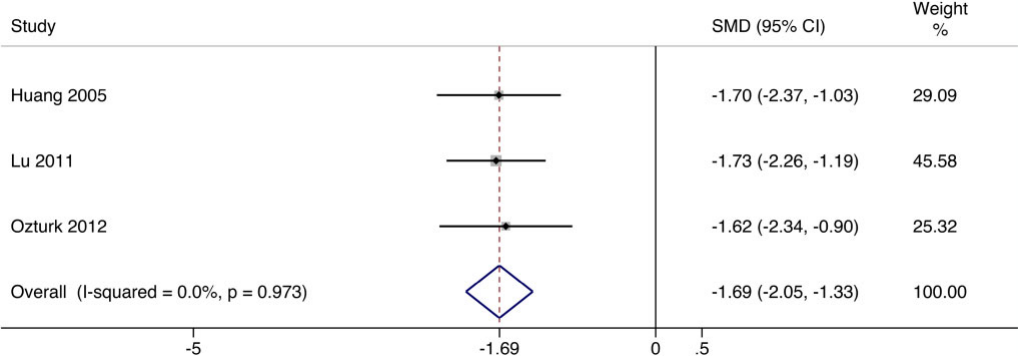
Forest plots for meta-analysis of pain scale comparing cellular therapy with standard therapy. Area of the symbols for each study (square) is proportional to study weight. The pooled standardized mean difference (fixed effects) and 95% confidence intervals are represented by the rhombus. Heterogeneity test across studies was significant (*I* ^2^ = 0.0%, *P* = 0.973). CI, confidence interval; SMD, standardized mean difference.

Three trials reported the pain scale of patients with diabetic foot ulcer. Ozturk et al.^18^ used numerical pain rating scale which ranged from 0 to 10, 0 being no pain and 10 being maximum pain.^22^ In the randomized controlled trials conducted by Lu et al.^16^ and Huang et al.,^15^ rest pain scores on rating scales ranged from 0 for the best (completely resolved) to 4 points for the worst condition (severe pain unresolved with paracetamol or nonsteroidal anti-inflammatory drugs).^22^ There was no heterogeneity among these studies (*I* ^2^ = 0.0%, *P* = 0.973). The pain scale was significantly decreased (SMD = −1.69, 95% CI = −2.05 to −1.33) in the selected studies in the cellular therapy group (Fig. 4).

### Ulcer Healing Rate

**Fig. 5.**
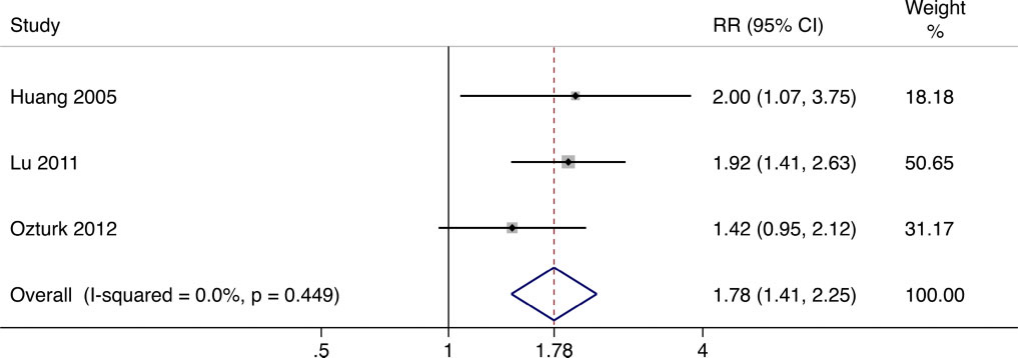
Forest plots for meta-analysis of ulcer healing rate comparing cellular therapy with standard therapy. Area of the symbols for each study (square) is proportional to study weight. The pooled relative risk (fixed effects) and 95% confidence intervals are represented by the rhombus. Heterogeneity test across studies was significant (*I* ^2^ = 0.0%, *P* = 0.449). CI, confidence interval; RR, relative risk.

With regard to the efficacy of cellular therapy in contrast to the standard therapy, the relative risk of 3 trials^15,16,18^ demonstrated a significant increase (relative risk [RR] = 1.78, 95% CI = 1.41 to 2.25) in ulcer healing rate 12 to 24 wk after cellular therapy using a fixed effects model (*I* ^2^ = 0.0%, *P* = 0.449; Fig. 5).

### AFS

**Fig. 6.**
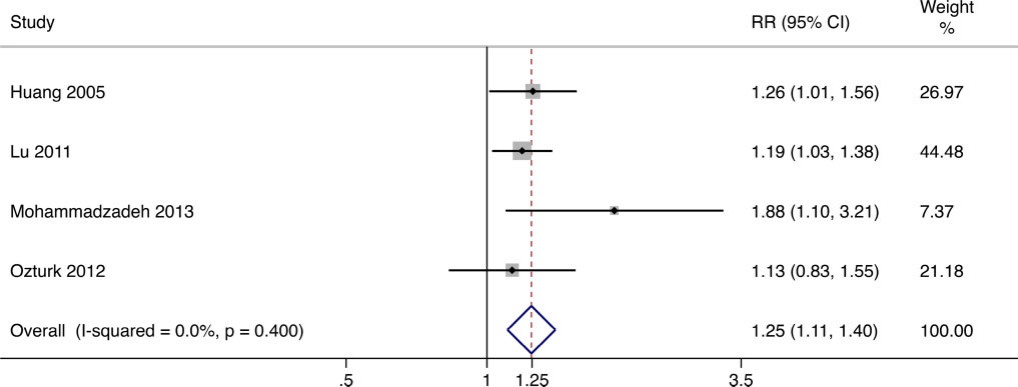
Forest plots for meta-analysis of amputation-free survival comparing cellular therapy with standard therapy. Area of the symbols for each study (square) is proportional to study weight. The pooled relative risk (fixed effects) and 95% confidence intervals are represented by the rhombus. Heterogeneity test across studies was significant (*I* ^2^ = 0.0%, *P* = 0.400). CI, confidence interval; RR, relative risk.

The combined end point of AFS was considered to be the best outcome assessment indexes for patients with diabetic foot ulcer.^23^ Four studies^15–18^ provided amputation data. With no heterogeneity (*I* ^2^ = 0.0%, *P* = 0.400), calculations under fixed effects model revealed that cellular therapy significantly improved the AFS rate in patients with diabetic foot ulcer (RR = 1.25, 95% CI = 1.11 to 1.40; Fig. 6).

### Adverse Events

During 1 to 2 y long-time follow-up, no serious complications resulted from cellular therapy, such as rejection, allergic reactions, and tumorigenesis were observed.^14–19^ No complications such as puncture site hematoma, pseudoaneurysm, arterial dissection, or cardiovascular or cerebrovascular events related to the transplantation procedure were detected.^14–19^ No infection, bleeding, or other complications arose from the microbiological condition of the cells were detected.^14–19^ Only 8 of 118 patients suffered from short-term episodes of slight pain after cell transplantation^15,16^ and 3 patients bled at the iliac crest after bone marrow aspiration.^15,16^

### Sensitivity Analysis

Sensitivity analysis was performed by reestimating the outcome by removing 1 study in each turn and was indicative of the reliability of the outcomes. Sensitivity analysis did not identify any marked difference in the direction and magnitude of the mean difference and relative risk with respect to ABI, TcPO_2,_ pain scale, ulcer healing rate, and AFS, indicating good reliability of the outcomes in this meta-analysis.

### Publication Bias

We assessed publication bias by Egger’s intercept test and Begg’s rank correlation analysis. The *P* values of the Egger’s test and Begg’s test were all greater than 0.05 for ABI (Begg’s test *P* = 1.000, Egger’s test *P* = 0.847), TcPO_2_ (Begg’s test *P* = 1.000, Egger’s test *P* = 0.549), pain scale (Begg’s test *P* = 0.296, Egger’s test *P* = 0.309), ulcer healing rate (Begg’s test *P* = 1.000, Egger’s test *P* = 0.975), and AFS (Begg’s test *P* = 0.308, Egger’s test *P* = 0.302), indicating no significant evidence of publication bias.

## Discussion

The purpose of the present study was to perform a meta-analysis to assess the efficacy and safety of cellular therapy in treatment of diabetic foot ulcer. Our study included 6 randomized controlled trials involving 241 patients and analyzed 5 end point indexes which were important in the long-term prognosis and quality of life of patients with diabetic foot ulcer. It was shown that cellular therapy was significantly associated with a higher ABI, a higher transcutaneous oxygen pressure, more reduction in pain, a decreased risk of amputation, and a higher proportion of healed ulcers, when compared with standard therapy. The complete ulcer healing rate and AFS in cellular therapy group is 1.78 and 1.25 times more than control group at 12 to 24 wk, respectively. These findings are robust, as sensitivity analysis had confirmed that deletion of any study would not change the direction of the outcomes. Possible security threats resulted from cellular therapy frighten some clinicians away. However, our pooled statistics showed that cellular therapy seemed to be safe, with no serious complications and low risk of short-term slight complications.

Cellular therapy is an attractive approach delivering self-renewing cells in regenerative medicine. In the recent years, researchers have utilized direct systemic administration, local intramuscular injection, or direct application over the wound to deliver cells in cellular therapy.^24^ Among all of the included studies in this meta-analysis, cells were delivered into the area surrounding the wound by means of muscle injection. Intramuscular injection^25,26^ into the wound periphery was found to be safer and more efficient than systemic administration in animal research models and clinical studies.^27^ The primary shortcomings of systemic delivery were cell trafficking and risk of malignancy resulted from wide distribution of exogenous cells, together with low intended location arrival rate.^28,29^ Moreover, appropriate scaffold such as fibrin sealants has shown its advantage in promoting wound healing in animals and humans.^30^ Possible mechanisms include confining cells, promoting engraftment, and maximizing their therapeutic effects.^31^

Owing to no ethical controversy and diverse sources, adult cells are more preferred in clinical practice. Preclinical outcomes and clinical researches utilizing adult cells already have revealed attractive therapeutic promise in treating diabetic foot. In this review, adult cells were utilized in all the included literatures, consisting of BMMSCs,^14,16^ umbilical cord-derived mesenchymal stem cells (UCMSCs),^19^ bone marrow-derived MNCs (BMMNCs),^16^ and peripheral blood MNCs (PBMNCs).^15,18^

There is growing evidence that possible therapeutic mechanisms behind MSCs may be paracrine secretion of growth factors and cytokines^32^ and their direct differentiation into vascular endothelial cells and skin components.^33–35^ Lots of researches believed that paracrine secretion played a greater role in diabetic wound healing. Numerous studies have proven the high-level secretion of various growth factors and cytokines, such as epidermal growth factor (EGF), basic fibroblast growth factor (bFGF), keratinocyte growth factor (KGF), insulin-like growth factor (IGF-1), transforming growth factor-β (TGF- β), vascular endothelial growth factor (VEGF), smooth muscle cell-derived growth factor-1α, interleukin-8 (IL-8), and angiopoietin-1.^36–38^ These growth factors and cytokines exert diverse therapeutic effects in diabetic wound healing including improving neovascularization, angiogenesis, regeneration, ameliorating inflammation, and interestingly, recruiting endogenous stem cells from the circulation for repair.^39^ Reyes et al. also observed that transplanted mesenchymal stem cells could be differentiated into angioblasts and vascular endothelial cells and then functioned as mature endotheliocytes, contributing to neoangiogenesis in diabetic foot ulcer model.^40^ However, differentiation may be of limited use as there is a low proportion of engraftment and differentiation.^35,41^

MNCs are a group of cells consisting of several stem/progenitor cell populations and some other cell types. They are so abundant in peripheral blood and bone marrow that they can be collected directly for transplantation with no need for in vitro expansion. MNCs were found to promote local capillary and blood vessel reconstitution in infarcted limbs in a study by Stamm et al.^42^ Sivan-Loukianova et al. reported accelerated epidermal healing and revascularization after MNC transplantation in a diabetic mouse wound healing model. During a 5 d observation period, an increase in vessel diameter was the main manifestation at early stages. Later, increases in vessel size and number both accounted for increased vascularization.^43^

Studies also showed that endothelial progenitor cells (EPCs) from bone marrow or peripheral blood could proliferate, migrate, and be mobilized under ischemic stimulation in some pathological conditions.^44,45^ Accumulating evidence has proven their therapeutic ability in diabetic foot ulcer.^46,47^ Wound healing promotion and neovascularization were found by using embryonic stem cell–derived EPCs in Lee’s study.^46^ They found rapid reepithelialization of wounds and reformation of granulation tissue after transplantation in a wound healing model. After further exploration, they put forward the idea that secretion of growth factors and cytokines by EPCs, including EGF, bFGF, VEGF, IL-6, IL-8, granulocyte-macrophage colony-stimulating factor (GM-CSF), and platelet-derived growth factor-AA (PDGF-AA), may account for the main therapeutic effect.^46^ EPCs display endothelial-like characteristics, and their neovascularization effect seemed to be particularly suited in improving microcirculation in the management of diabetic foot ulcer. Taking the features of directed migration and vascularization into consideration comprehensively, EPCs not only play a part in tissue repair but also brought reperfusion into ischemic regions.^48^ Asahara et al. reported enhanced capillary density and recovery of blood flow after EPC transplantation in athymic nude mice with hind limb ischemia.^45^

As far as we know, this study is the first meta-analysis of randomized controlled studies comparing the clinical efficacy of cellular therapy in the management of diabetic foot ulcer. Limitations still existed in this study. First, the number of the included randomized controlled trials was small. Although there has been a growing number of studies reporting cellular therapy in diabetic foot ulcer, only a few studies fully meet our requirements. Second, the sample size and quality of the included studies remained a concern for the strength of the outcomes. Only 1 study contains more than 30 participants in each group. And information of allocation concealment was only reported in 1 study. Third was the variability of measurements and criterion of ulcer healing. Forth, baseline ulcer conditions that would affect the outcomes were not reported in all of the studies.

## Conclusions

Compared to standard therapy, cellular therapy could help accelerate the healing of diabetic foot ulcer, which presents as higher ABI, TcPO_2_, ulcer healing rate, and lower scale of pain and amputation risk. These results need to be treated with caution, as the number of available randomized controlled studies and the follow-up duration were limited. More large-scale, well-designed randomized controlled studies with long follow-up duration are in urgent need to further examine the clinical value of cellular therapy in the management of diabetic foot ulcer.

## References

[1] Whiting DR Guariguata L Weil C Shaw J IDF diabetes atlas: global estimates of the prevalence of diabetes for 2011 and 2030. Diabetes Res Clin Pract. 2011;94(3):311–321. 22079683 10.1016/j.diabres.2011.10.029

[2] Boulton AJ The pathway to foot ulceration in diabetes. Med Clin North Am. 2013;97(5):775–790. 23992891 10.1016/j.mcna.2013.03.007

[3] Singh N Armstrong DG Lipsky BA Preventing foot ulcers in patients with diabetes. JAMA. 2005;293(2):217–228. 15644549 10.1001/jama.293.2.217

[4] Frykberg RG Armstrong DG Giurini J Edwards A Kravette M Kravitz S Ross C Stavosky J Stuck R Vanore J Diabetic foot disorders: a clinical practice guideline. American College of Foot and Ankle Surgeons. J Foot Ankle Surg. 2000;39(5 suppl):S1–S60. 11280471

[5] Eldor R Raz I Ben Yehuda A Boulton AJ New and experimental approaches to treatment of diabetic foot ulcers: a comprehensive review of emerging treatment strategies. Diabet Med. 2004;21(11):1161–1173. 15498081 10.1111/j.1464-5491.2004.01358.x

[6] Brod M Nikolajsen A Weatherall J Pfeiffer KM The economic burden of post-prandial hyperglycemia (PPH) among people with type 1 and type 2 diabetes in three countries. Diabetes Ther. 2016;7(1):75–90. 26899431 10.1007/s13300-016-0154-2 PMC4801810

[7] O’Loughlin A McIntosh C Dinneen SF O’Brien T Review paper: basic concepts to novel therapies: a review of the diabetic foot. Int J Low Extrem Wounds. 2010;9(2):90–102. 20483808 10.1177/1534734610371600

[8] Cavanagh PR Bus SA Off-loading the diabetic foot for ulcer prevention and healing. Plast Reconstr Surg. 2011;127(suppl 1):248s–256s. 21200298 10.1097/PRS.0b013e3182024864

[9] Cavanagh PR Lipsky BA Bradbury AW Botek G Treatment for diabetic foot ulcers. Lancet. 2005;366(9498):1725–1735. 16291067 10.1016/S0140-6736(05)67699-4

[10] Lipsky BA Berendt AR Deery HG Embil JM Joseph WS Karchmer AW LeFrock JL Lew DP Mader JT Norden C Diagnosis and treatment of diabetic foot infections. Plast Reconstr Surg. 2006;117(7 Suppl):212s–238s. 16799390 10.1097/01.prs.0000222737.09322.77

[11] Yang M Sheng L Zhang TR Li Q Stem cell therapy for lower extremity diabetic ulcers: where do we stand? Biomed Res Int. 2013;2013:462179. 10.1155/2013/462179 PMC3613085 23586040

[12] Blumberg SN Berger A Hwang L Pastar I Warren SM Chen W The role of stem cells in the treatment of diabetic foot ulcers. Diabetes Res Clin Pract. 2012;96(1):1–9. 22142631 10.1016/j.diabres.2011.10.032

[13] Jadad AR Moore RA Carroll D Jenkinson C Reynolds DJ Gavaghan DJ McQuay HJ Assessing the quality of reports of randomized clinical trials: is blinding necessary? Control Clin Trials. 1996;17(1):1–12. 8721797 10.1016/0197-2456(95)00134-4

[14] Dash NR Dash SN Routray P Mohapatra S Mohapatra PC Targeting nonhealing ulcers of lower extremity in human through autologous bone marrow-derived mesenchymal stem cells. Rejuvenation Res. 2009;12(5):359–366. 19929258 10.1089/rej.2009.0872

[15] Huang P Li S Han M Xiao Z Yang R Han ZC Autologous transplantation of granulocyte colony-stimulating factor-mobilized peripheral blood mononuclear cells improves critical limb ischemia in diabetes. Diabetes Care. 2005;28(9):2155–2160. 16123483 10.2337/diacare.28.9.2155

[16] Lu D Chen B Liang Z Deng W Jiang Y Li S Xu J Wu Q Zhang Z Xie B Comparison of bone marrow mesenchymal stem cells with bone marrow-derived mononuclear cells for treatment of diabetic critical limb ischemia and foot ulcer: a double-blind, randomized, controlled trial. Diabetes Res Clin Pract. 2011;92(1):26–36. 21216483 10.1016/j.diabres.2010.12.010

[17] Mohammadzadeh L Samedanifard SH Keshavarzi A Alimoghaddam K Larijani B Ghavamzadeh A Ahmadi AS Shojaeifard A Ostadali MR Sharifi AM Therapeutic outcomes of transplanting autologous granulocyte colony-stimulating factor-mobilised peripheral mononuclear cells in diabetic patients with critical limb ischaemia. Exp Clin Endocrinol Diabetes. 2013;121(1):48–53. 23329572 10.1055/s-0032-1311646

[18] Ozturk A Kucukardali Y Tangi F Erikci A Uzun G Bashekim C Sen H Terekeci H Narin Y Ozyurt M Therapeutical potential of autologous peripheral blood mononuclear cell transplantation in patients with type 2 diabetic critical limb ischemia. J Diabetes Complications. 2012;26(1):29–33. 22240264 10.1016/j.jdiacomp.2011.11.007

[19] Qin HL Zhu XH Zhang B Zhou L Wang WY Clinical evaluation of human umbilical cord mesenchymal stem cell transplantation after angioplasty for diabetic foot. Exp Clin Endocrinol Diabetes. 2016;124(8):497–503. 27219884 10.1055/s-0042-103684

[20] Cao P Eckstein HH De Rango P Setacci C Ricco JB de Donato G Becker F Robert-Ebadi H Diehm N Schmidli J Chapter II: diagnostic methods. Eur J Vasc Endovasc Surg. 2011;42(Suppl 2):S13–S32. 22172470 10.1016/S1078-5884(11)60010-5

[21] Poredos P Rakovec S Guzic-Salobir B Determination of amputation level in ischaemic limbs using tcPO2 measurement. Vasa. 2005;34(2):108–112. 15968892 10.1024/0301-1526.34.2.108

[22] Williamson A Hoggart B Pain: a review of three commonly used pain rating scales. J Clin Nurs. 2005;14(7):798–804. 16000093 10.1111/j.1365-2702.2005.01121.x

[23] Chung J Timaran DA Modrall JG Ahn C Timaran CH Kirkwood ML Baig MS Valentine RJ Optimal medical therapy predicts amputation-free survival in chronic critical limb ischemia. J Vasc Surg. 2013;58(4):972–980. 23993439 10.1016/j.jvs.2013.03.050

[24] Badiavas EV Falanga V Treatment of chronic wounds with bone marrow-derived cells. Arch Dermatol. 2003;139(4):510–516. 12707099 10.1001/archderm.139.4.510

[25] Humpert PM Bartsch U Konrade I Hammes HP Morcos M Kasper M Bierhaus A Nawroth PP Locally applied mononuclear bone marrow cells restore angiogenesis and promote wound healing in a type 2 diabetic patient. Exp Clin Endocrinol Diabetes. 2005;113(9):538–540. 16235157 10.1055/s-2005-872886

[26] Rogers LC Bevilacqua NJ Armstrong DG The use of marrow-derived stem cells to accelerate healing in chronic wounds. Int Wound J. 2008;5(1):20–25. 18179555 10.1111/j.1742-481X.2007.00349.x PMC7951309

[27] Jiang XY Lu DB Chen B Progress in stem cell therapy for the diabetic foot. Diabetes Res Clin Pract. 2012;97(1):43–50. 22221581 10.1016/j.diabres.2011.12.011

[28] Lobmann R Schultz G Lehnert H Proteases and the diabetic foot syndrome: mechanisms and therapeutic implications. Diabetes Care. 2005;28(2):461–471. 15677818 10.2337/diacare.28.2.461

[29] Yager DR Nwomeh BC The proteolytic environment of chronic wounds. Wound Repair Regen. 1999;7(6):433–441. 10633002 10.1046/j.1524-475x.1999.00433.x

[30] Falanga V Iwamoto S Chartier M Yufit T Butmarc J Kouttab N Shrayer D Carson P Autologous bone marrow-derived cultured mesenchymal stem cells delivered in a fibrin spray accelerate healing in murine and human cutaneous wounds. Tissue Eng. 2007;13(6):1299–1312. 17518741 10.1089/ten.2006.0278

[31] Gnecchi M Zhang Z Ni A Dzau VJ Paracrine mechanisms in adult stem cell signaling and therapy. Circ Res. 2008;103(11):1204–1219. 19028920 10.1161/CIRCRESAHA.108.176826 PMC2667788

[32] Peng C Chen B Kao HK Murphy G Orgill DP Guo L Lack of FGF-7 further delays cutaneous wound healing in diabetic mice. Plast Reconstr Surg. 2011;128(6):673e–684e. 10.1097/PRS.0b013e318230c521 22094769

[33] Kim SW Zhang HZ Guo L Kim JM Kim MH Amniotic mesenchymal stem cells enhance wound healing in diabetic NOD/SCID mice through high angiogenic and engraftment capabilities. PLoS One. 2012;7(7):e41105. 22815931 10.1371/journal.pone.0041105 PMC3398889

[34] Wu Y Chen L Scott PG Tredget EE Mesenchymal stem cells enhance wound healing through differentiation and angiogenesis. Stem Cells. 2007;25(10):2648–2659. 17615264 10.1634/stemcells.2007-0226

[35] Uysal CA Ogawa R Lu F Hyakusoku H Mizuno H Effect of mesenchymal stem cells on skin graft to flap prefabrication: an experimental study. Ann Plast Surg. 2010;65(2):237–244. 20585233 10.1097/SAP.0b013e3181c1ff14

[36] Usui ML Mansbridge JN Carter WG Fujita M Olerud JE Keratinocyte migration, proliferation, and differentiation in chronic ulcers from patients with diabetes and normal wounds. J Histochem Cytochem. 2008;56(7):687–696. 18413645 10.1369/jhc.2008.951194 PMC2430161

[37] Desta T Li J Chino T Graves DT Altered fibroblast proliferation and apoptosis in diabetic gingival wounds. J Dent Res. 2010;89(6):609–614. 20354230 10.1177/0022034510362960 PMC3318033

[38] Loot MA Kenter SB Au FL van Galen WJ Middelkoop E Bos JD Mekkes JR Fibroblasts derived from chronic diabetic ulcers differ in their response to stimulation with EGF, IGF-I, bFGF and PDGF-AB compared to controls. Eur J Cell Biol. 2002;81(3):153–160. 11998867 10.1078/0171-9335-00228

[39] Chen L Tredget EE Wu PY Wu Y Paracrine factors of mesenchymal stem cells recruit macrophages and endothelial lineage cells and enhance wound healing. PLoS One. 2008;3(4):e1886. 18382669 10.1371/journal.pone.0001886 PMC2270908

[40] Reyes M Dudek A Jahagirdar B Koodie L Marker PH Verfaillie CM Correction: origin of endothelial progenitors in human postnatal bone marrow. J Clin Invest. 2008;118(11):3813. 27809420 10.1172/JCI14327C1 PMC2575714

[41] Lee SH Jin SY Song JS Seo KK Cho KH Paracrine effects of adipose-derived stem cells on keratinocytes and dermal fibroblasts. Ann Dermatol. 2012;24(2):136–143. 22577262 10.5021/ad.2012.24.2.136 PMC3346902

[42] Stamm C Westphal B Kleine HD Petzsch M Kittner C Klinge H Schumichen C Nienaber CA Freund M Steinhoff G Autologous bone-marrow stem-cell transplantation for myocardial regeneration. Lancet. 2003;361(9351):45–46. 12517467 10.1016/S0140-6736(03)12110-1

[43] Sivan-Loukianova E Awad OA Stepanovic V Bickenbach J Schatteman GC CD34+ blood cells accelerate vascularization and healing of diabetic mouse skin wounds. J Vasc Res. 2003;40(4):368–377. 12891006 10.1159/000072701

[44] Deng X Szabo S Chen L Paunovic B Khomenko T Tolstanova G Tarnawski AS Jones MK Sandor Z New cell therapy using bone marrow-derived stem cells/endothelial progenitor cells to accelerate neovascularization in healing of experimental ulcerative colitis. Curr Pharm Des. 2011;17(16):1643–1651. 21548863 10.2174/138161211796197007

[45] Asahara T Masuda H Takahashi T Kalka C Pastore C Silver M Kearne M Magner M Isner JM Bone marrow origin of endothelial progenitor cells responsible for postnatal vasculogenesis in physiological and pathological neovascularization. Circ Res. 1999;85(3):221–228. 10436164 10.1161/01.res.85.3.221

[46] Lee MJ Kim J Lee KI Shin JM Chae JI Chung HM Enhancement of wound healing by secretory factors of endothelial precursor cells derived from human embryonic stem cells. Cytotherapy. 2011;13(2):165–178. 21235296 10.3109/14653249.2010.512632

[47] Park S Tepper OM Galiano RD Capla JM Baharestani S Kleinman ME Pelo CR Levine JP Gurtner GC Selective recruitment of endothelial progenitor cells to ischemic tissues with increased neovascularization. Plast Reconstr Surg. 2004;113(1):284–293. 14707648 10.1097/01.PRS.0000091169.51035.A5

[48] Schatteman GC Dunnwald M Jiao C Biology of bone marrow-derived endothelial cell precursors. Am J Physiol Heart Circ Physiol. 2007;292(1):H1–H18. 16980351 10.1152/ajpheart.00662.2006

